# Supplementary material for: Compliance with Iron and Folic Acid Supplementation (IFAS) and associated factors among pregnant women in Sub-Saharan Africa: A systematic review and meta-analysis
Source: PLoS One. 2021 Apr 14;16(4):e0249789. doi: 10.1371/journal.pone.0249789 (PMC8046188; doi:10.1371/journal.pone.0249789)
Supplement: S1 Fig — Description of figure: This figure presents, Bag’s and Egger’s test for publication bias showed no statistical evidence of publication bias. (DOCX) [file pone.0249789.s001.docx]

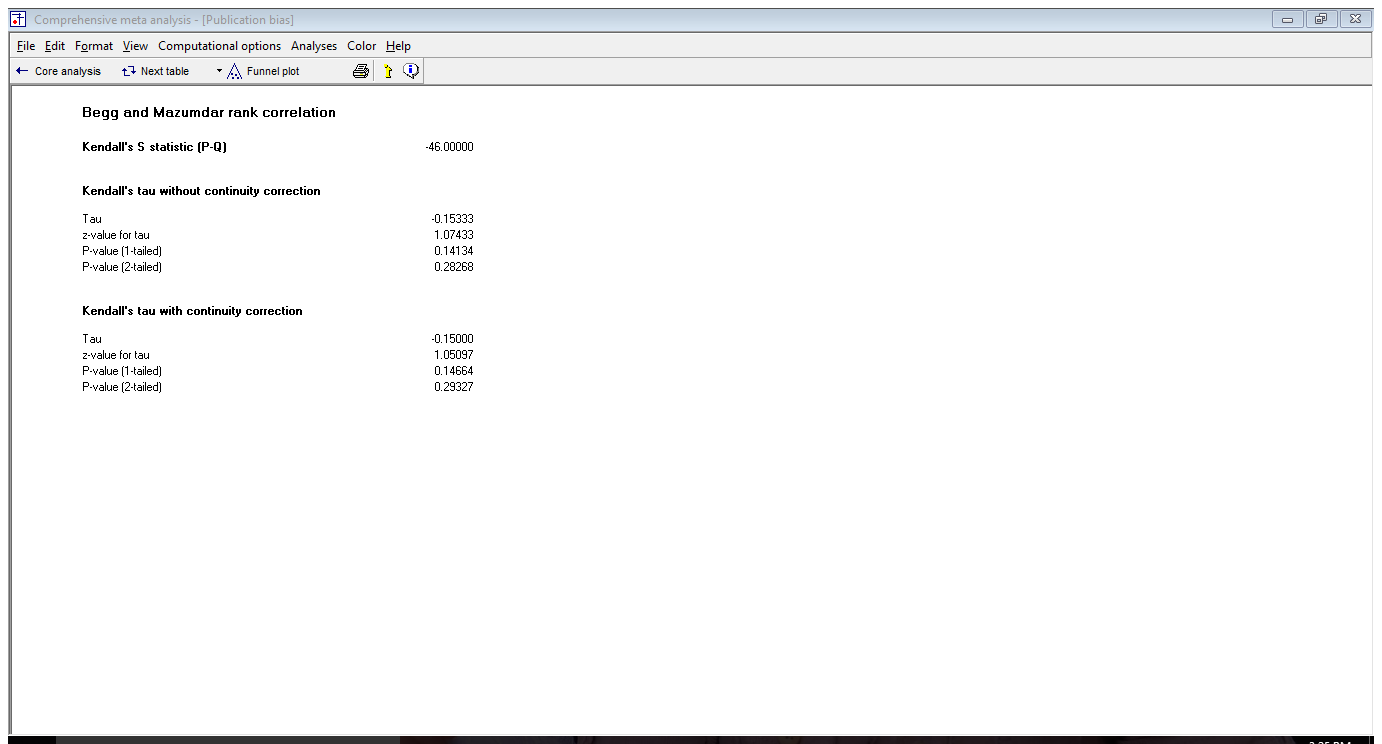


**S1 Fig. Funnel plot displaying publication bias of prevalence of compliance with IFAS among pregnant women in Sub-Saharan Africa.**
