# Supplementary material for: Compliance with Iron and Folic Acid Supplementation (IFAS) and associated factors among pregnant women in Sub-Saharan Africa: A systematic review and meta-analysis
Source: PLoS One. 2021 Apr 14;16(4):e0249789. doi: 10.1371/journal.pone.0249789 (PMC8046188; doi:10.1371/journal.pone.0249789)
Supplement: S2 File — (DOCX) [file pone.0249789.s003.docx]

  Result of Quality assessment Using Modified Newcastle – Ottawa scale

The total score for the modified Newcastle – Ottawa scale for cross sectional studies is nine (9) stars as a maximum for the overall scale with the minimum of zero. A study was considered high quality if it achieved 7 out 9 and medium if it achieved 5 out of 9 (46).

| Studies | Representativeness of the sample | | | Sample size | | | Non-respondents | Ascertainment of the exposure (risk factor) | | | Confounding factors are controlled | | Assessment of the outcome | Statistical test | | | | Total |  |
| --- | --- | --- | --- | --- | --- | --- | --- | --- | --- | --- | --- | --- | --- | --- | --- | --- | --- | --- | --- |
|  | All subjects or random sampling * | Non random sampling * | No description of sampling strategy | Justified and satisfactory * | Not Justified | Satisfactory * | Unsatisfactory | Validated measurement tool ** | non Validated measure or tool is available * | No description of Validated measurement tool | Study controls important factor * | Study controls Additional factor * | Independent blind assessment ** | Record linkage ** | Self-report * | No description | statistical test used to analyze the data is clearly described* |  |  |
| Abinet Arega Sadore et al,2015 | * |  |  | * |  | * |  | * |  |  |  |  | ** |  |  |  |  | 6 | |
| Titilayo A.et al,2016 | * |  |  | * |  | * |  | * |  |  | * |  | ** |  |  |  |  |  | |
| Chikakuda A. et al,2018 | * |  |  | * |  | * |  | ** |  |  | * |  | ** |  |  |  |  | 8 | |
| Bekele Taye et al,2015 | * |  |  | * |  | * |  | ** |  |  |  |  | * |  |  |  | * | 7 | |
| BI Nwaru et al,2014 | * |  |  | * |  |  |  | ** |  |  | * |  | * |  |  |  | * |  | |
| Demis et al,2019 | * |  |  | * |  | * |  | ** |  |  | * |  | ** |  |  |  | * | 9 | |
| Agegnehu G. et al,2018 | * |  |  | * |  |  |  | ** |  |  | * |  | ** |  |  |  | * | 8 | |
| Gebremariam et al,2019 | * |  |  | * |  | * |  | * |  |  | * |  | ** |  |  |  | * | 8 | |
| Getachew et al, 2018 | * |  |  | * |  | * |  | * |  |  | * |  | * |  |  |  | * |  | |
| Dessie G. et al,2018 | * |  |  | * |  | * |  | * |  |  | * |  | ** |  |  |  | * |  | |
| Juma M et al,2015 | * |  |  |  |  | * |  | * |  |  | * |  | ** |  |  |  | * | 7 | |
| K. Niang et al,2017 | * |  |  | * |  | * |  | ** |  |  | * |  | ** |  |  |  | * |  | |
| Kamau et al,2018 | * |  |  | * |  | * |  | ** |  |  | * |  | ** |  |  |  | * | 9 | |
| Kiwanuka et al,2017 | * |  |  | * |  | * |  | ** |  |  | * |  | ** |  |  |  |  | 8 | |
| Lucy Nyandia Gathigi,2011 | * |  |  | * |  | * |  | ** |  |  | * |  | ** |  |  |  | * |  | |
| LYNETTE AOKO DINGA,2013 | * |  |  | * |  |  |  | ** |  |  | * |  | * |  |  |  | * | 7 | |
| Mbhenyane et al,2017 | * |  |  | * |  | * |  | ** |  |  | * |  | ** |  |  |  | * |  | |
| Molla et al,2019 | * |  |  | * |  | * |  | ** |  |  | * |  | * |  |  |  | * | 8 | |
| Niguse and Murugan,2018 | * |  |  | * |  | * |  | ** |  |  | * |  | ** |  |  |  | * | 9 | |
| Onyeneho et al,2016 | * |  |  |  |  | * |  | * |  |  | * |  | ** |  |  |  | * | 6 | |
| Shewasinad S, et al,2017 | * |  |  | * |  | * |  | ** |  |  | * |  | ** |  |  |  | * | 9 | |
| Tarekegn et al,2019 | * |  |  | * |  | * |  | ** |  |  | * |  | ** |  |  |  |  | 8 | |
| Ugwu, et al,2012 | * |  |  | * |  | * |  | ** |  |  | * |  | ** |  |  |  | * | 9 | |
