# Supplementary material for: Compliance with Iron and Folic Acid Supplementation (IFAS) and associated factors among pregnant women in Sub-Saharan Africa: A systematic review and meta-analysis
Source: PLoS One. 2021 Apr 14;16(4):e0249789. doi: 10.1371/journal.pone.0249789 (PMC8046188; doi:10.1371/journal.pone.0249789)
Supplement: S3 File — (DOCX) [file pone.0249789.s004.docx]

1. Data Extraction
2. Characteristics of studies

| Author | Region | Study design | Study type | Sample Size | Prevalence in % | Compliance IFAS | No compliance IFAS | Citation ( Reference number) |
| --- | --- | --- | --- | --- | --- | --- | --- | --- |
| Abinet Arega Sadore et al,2015 | Ethiopia | Cross sectional | Community based | 296 | 39.2 | 116 | 180 | 25 |
| Titilayo A.et al,2016 | Malawi | Cross sectional | Community based | 10750 | 37.2 | 3999 | 6751 | 23 |
| Chikakuda A. et al,2018 | Malawi | Cross sectional | Facility based | 213 | 18.3 | 39 | 174 | 24 |
| Bekele Taye et al,2015 | Ethiopia | Cross sectional | Community based | 628 | 20.4 | 128 | 500 | 26 |
| BI Nwaru et al,2014 | Mozambique | Cross sectional | Facility based | 4326 | 79 | 3418 | 908 | 27 |
| Demis et al,2019 | Ethiopia | Cross sectional | Facility based | 422 | 43.1 | 182 | 240 | 28 |
| Agegnehu G. et al,2018 | Ethiopia | Cross sectional | Facility based | 418 | 28.7 | 120 | 298 | 29 |
| Gebremariam et al,2019 | Ethiopia | Cross sectional | Facility based | 241 | 40 | 96 | 145 | 30 |
| Getachew et al, 2018 | Ethiopia | Cross sectional | Facility based | 320 | 64.7 | 207 | 113 | 31 |
| Dessie G. et al,2018 | Ethiopia | Cross sectional | Facility based | 348 | 19 | 66 | 282 | 32 |
| Juma M et al,2015 | Kenya | Cross sectional | Facility based | 352 | 18.3 | 64 | 288 | 33 |
| K. Niang et al,2017 | Senegal | Cross sectional | Community based | 1442 | 51 | 735 | 707 | 34 |
| Kamau et al,2018 | Kenya | Cross sectional | Facility based | 364 | 33.7 | 123 | 241 | 35 |
| Kiwanuka et al,2017 | Uganda | Cross sectional | Facility based | 370 | 11.6 | 43 | 327 | 36 |
| Lucy Nyandia Gathigi,2011 | Kenya | Cross sectional | Facility based | 264 | 10.6 | 28 | 236 | 37 |
| LYNETTE AOKO DINGA,2013 | Kenya | Cross sectional | Facility based | 200 | 24.5 | 49 | 151 | 38 |
| Mbhenyane et al,2017 | South Africa | Cross sectional | Community based | 57 | 90 | 51 | 6 | 39 |
| Molla et al,2019 | Ethiopia | Cross sectional | Facility based | 348 | 52.9 | 184 | 164 | 40 |
| Niguse and Murugan,2018 | Ethiopia | Cross sectional | Facility based | 296 | 59.8 | 177 | 119 | 41 |
| Onyeneho et al,2016 | Nigeria | Cross sectional | Facility based | 1500 | 33 | 495 | 1005 | 42 |
| Shewasinad S, et al,2017 | Ethiopia | Cross sectional | Facility based | 326 | 70.6 | 230 | 96 | 45 |
| Tarekegn et al,2019 | Ethiopia | Cross sectional | Facility based | 395 | 28.0 | 111 | 284 | 43 |
| Ugwu, et al,2012 | Nigeria | Cross sectional | Facility based | 396 | 65.9 | 261 | 135 | 44 |

1. Knowledge of IFAS

| Author name | Sample Size | Have Knowledge of IFAS | Total | No Knowledge of IFAS | Total |
| --- | --- | --- | --- | --- | --- |
| Abinet Arega Sadore et al,2015 | 296 | 101 | 182 | 15 | 114 |
| Bekele Taye et al,2015 | 628 | 75 | 156 | 53 | 472 |
| Demis et al,2019 | 422 | 139 | 242 | 43 | 180 |
| Gebremariam et al,2019 | 241 | 71 | 124 | 31 | 112 |
| Dessie G. et al,2018 | 342 | 52 | 79 | 111 | 238 |
| Kamau et al,2018 | 364 | 73 | 149 | 46 | 169 |
| Kassa et al,2019 | 402 | 120 | 203 | 34 | 199 |
| Lucy Nyandia Gathigi,2011 | 264 | ***45*** | 50 | 172 | 214 |
| LYNETTE AOKO DINGA,2013 | 200 | 26 | 69 | 23 | 131 |
| Molla et al,2019 | 348 | 102 | 239 | 82 | 109 |

1. Knowledge of Anemia

| Author name | Sample Size | Have Knowledge of Anemia | Total | No Knowledge Anemia | Total |
| --- | --- | --- | --- | --- | --- |
| Abinet Arega Sadore et al,2015 | 296 | 101 | 182 | 15 | 114 |
| Bekele Taye et al,2015 | 628 | 75 | 156 | 53 | 472 |
| Demis et al,2019 | 422 | 139 | 242 | 43 | 180 |
| Gebremariam et al,2019 | 241 | 71 | 124 | 31 | 112 |
| Dessie G. et al,2018 | 342 | 52 | 79 | 111 | 238 |
| Kamau et al,2018 | 364 | 73 | 149 | 46 | 169 |
| Kassa et al,2019 | 402 | 120 | 203 | 34 | 199 |
| Lucy Nyandia Gathigi,2011 | 264 | ***45*** | 50 | 172 | 214 |
| LYNETTE AOKO DINGA,2013 | 200 | 26 | 69 | 23 | 131 |
| Molla et al,2019 | 348 | 102 | 239 | 82 | 109 |

1. Fourth visit of ANC

| Author name | Sample Size | Have ANC4 Visit | Total | No ANC4 Visit | Total |
| --- | --- | --- | --- | --- | --- |
| Abinet Arega Sadore et al,2015 | 296 | 26 | 35 | 90 | 261 |
| Demis et al,2019 | 422 | 44 | 59 | 138 | 363 |
| Gebremariam et al,2019 | 241 | 86 | 156 | 21 | 85 |
| Agegnehu G. et al 2017 | 320 | 16 | 88 | 97 | 232 |
| Kiwanuka et al,2017 | 370 | 23 | 148 | 20 | 222 |
| LYNETTE AOKO DINGA,2013 | 200 | 15 | 50 | 33 | 150 |
| Molla et al,2019 | 348 | 13 | 62 | 171 | 286 |
| Tarekegn et al,2019 | 395 | 43 | 109 | 64 | 273 |

1. Counseling on IFAS

| Author name | Sample Size | Counseled on IFASA | Total | Not Counseled on IFASA | Total |
| --- | --- | --- | --- | --- | --- |
| Abinet Arega Sadore et al,2015 | 296 | 88 | 154 | 28 | 142 |
| Demis et al,2019 | 422 | 169 | 352 | 158 | 344 |
| Gebremariam et al,2019 | 241 | 52 | 94 | 55 | 92 |
| Getachew et al, 2018 | 320 | 42 | 181 | 71 | 139 |
| Kamau et al,2018 | 364 | 73 | 149 | 46 | 169 |
| Kiwanuka et al,2017 | 370 | 28 | 132 | 18 | 238 |
| Lucy Nyandia Gathigi,2011 | 264 | 34 | 36 | 175 | 228 |
| LYNETTE AOKO DINGA,2013 | 200 | 28 | 69 | 23 | 131 |
| Shewasinad S, et al,2017 | 326 | 303 | 414 | 22 | 48 |
