# Supplementary material for: Compliance with Iron and Folic Acid Supplementation (IFAS) and associated factors among pregnant women in Sub-Saharan Africa: A systematic review and meta-analysis
Source: PLoS One. 2021 Apr 14;16(4):e0249789. doi: 10.1371/journal.pone.0249789 (PMC8046188; doi:10.1371/journal.pone.0249789)
Supplement: Attachment — Submitted filename: A rebuttal letter.docx [file pone.0249789.s005.docx]

Editors

PLOS ONE

0ctober 8, 2020

Meseret Belete Fite

We thank you and the reviewers for a thorough reading and constructive criticism of our manuscript and for the opportunity to revise and resubmit. We are pleased to submit the improved research article Compliance with Iron and Folic Acid Supplementation (IFAS) And Associated Factors Among Pregnant Women Sub-Saharan Africa: A Systematic Review ” for your consideration in PLOS ONE. On the following pages, you will find our response to the editor and reviewer comments. On behalf of my co-authors, I thank you for your consideration of this resubmission. We appreciate your time and look forward to your response.

Meseret Belete Fite

Journal requirements:

1. Please ensure that your manuscript meets PLOS ONE's style requirements, including those for file naming. The PLOS ONE style templates can be found at

<https://journals.plos.org/plosone/s/file?id=wjVg/PLOSOne_formatting_sample_main_body.pdf> and

<https://journals.plos.org/plosone/s/file?id=ba62/PLOSOne_formatting_sample_title_authors_affiliations.pdf>

  -We unsure our manuscript meets PLOS ONE's style requirements, including those for file naming.

2. Please confirm that you have included all items recommended in the PRISMA checklist including the full electronic search strategy used to identify studies with all search terms and limits for at least one database. PLOS ONE's style requirements, including those for file naming.

- We have confirmed that we have included all items recommended in the PRISMA checklist including the full electronic search strategy used to identify

3. Please ensure that you refer to Figure 6 in your text as, if accepted, production will need this reference to link the reader to the figure.

  - We have checked Figure 6 in your text and corrected it.

4. Please upload a copy of Figure 4, to which you refer in your text on page 5. If the figure is no longer to be included as part of the submission please remove all reference to it within the text. (Fig 5 2x)

 - We have uploaded figure 4.

5. Thank you for submitting the above manuscript to PLOS ONE. During our internal evaluation of the manuscript, we found significant text overlap between your submission and the following previously published works.

- <https://bmchematol.biomedcentral.com/track/pdf/10.1186/s12878-018-0124-1>
- <https://archpublichealth.biomedcentral.com/articles/10.1186/s13690-019-0356-y?optIn=false>

We would like to make you aware that copying extracts from previous publications, especially outside the methods section, word-for-word is unacceptable. In addition, the reproduction of text from published reports has implications for the copyright that may apply to the publications.

Please revise the manuscript to rephrase the duplicated text, cite your sources, and provide details as to how the current manuscript advances on previous work. Please note that further consideration is dependent on the submission of a manuscript that addresses these concerns about the overlap in text with published work.

We will carefully review your manuscript upon resubmission, so please ensure that your revision is thorough.

**Author’s Responses Reviewers' comments**

Reviewers' comments:

Reviewer's Responses to Questions

**Comments to the Author**
1. Is the manuscript technically sound, and do the data support the conclusions?

The manuscript must describe a technically sound piece of scientific research with data that supports the conclusions. Experiments must have been conducted rigorously, with appropriate controls, replication, and sample sizes. The conclusions must be drawn appropriately based on the data presented.

Reviewer #1: Partly

Reviewer #2: Yes

2. Has the statistical analysis been performed appropriately and rigorously?

Reviewer #1: Yes

Reviewer #2: Yes

3. Have the authors made all data underlying the findings in their manuscript fully available?

The PLOS Data policy requires authors to make all data underlying the findings described in their manuscript fully available without restriction, with rare exception (please refer to the Data Availability Statement in the manuscript PDF file). The data should be provided as part of the manuscript or its supporting information, or deposited to a public repository. For example, in addition to summary statistics, the data points behind means, medians and variance measures should be available. If there are restrictions on publicly sharing data—e.g. participant privacy or use of data from a third party—those must be specified.

Reviewer #1: Yes

Reviewer #2: Yes

4. Is the manuscript presented in an intelligible fashion and written in standard English?

PLOS ONE does not copyedit accepted manuscripts, so the language in submitted articles must be clear, correct, and unambiguous. Any typographical or grammatical errors should be corrected at revision, so please note any specific errors here.

Reviewer #1: Yes

Reviewer #2: No

5. Review Comments to the Author

Please use the space provided to explain your answers to the questions above. You may also include additional comments for the author, including concerns about dual publication, research ethics, or publication ethics. (Please upload your review as an attachment if it exceeds 20,000 characters)

Reviewer #1: It was interesting study where authors summarized the compliance with Iron and folic acid supplementation (IFAS) and potential associated factors among pregnant women in Africa. Authors used meta-analysis to summarize this compliance, so results were valuable to some extent. This summary may be important for policy makers of Africa especially for maternal and child health care. However, some technical issues should be further addressed, which could improve the manuscript.
1. Due to limited studies included, the conclusion from this study was cautious. And studies included in analysis from one African country were limited (most of studies were form Ethiopia) and might be not a representative analysis for this country. Authors should deeply analyze this issue in the discussion.

We appreciate the efforts by you and the reviewers on the manuscript. We appreciate Reviewer #1 suggestion about the representativeness of the study. Considering your point, we have explained issues at the end paragraph of the discussion part

2. I think this was a meta-analysis, not a complete systematic review because lots of studies from different designs were excluded. Authors just made a mathematic analysis based on limited data. So I suggest that “A systematic review” in the title might be deleted.

Great point. We have changed the title:- **Compliance with Iron and Folic Acid Supplementation (IFAS) And Associated Factors Among Pregnant Women Sub-Saharan Africa: A Systematic Review.**

3. In the section of methods, authors said in line 121-222 that “Secondary outcome include: Counseling on IFAS, knowledge of IFA supplementation, knowledge of anaemia, fourth visit for ANC and early registration for ANC”. I disagree with it. Actually, authors still studied on primary outcome----a compliance of IFAS during pregnancy. This was further analysis about potential factors affecting the compliance of IFAS during pregnancy. Therefore, this sentence should be revised.

We appreciate your suggestion. As your argument, we have revised sentence “Study of Potential factors affecting the compliance of IFA supplementation comprise: Counseling on IFAS, knowledge of IFA supplementation, knowledge of anaemia, fourth visit for ANC and early registration for ANC was also Outcome interests of the current study”.

4. Authors should report the definition of Counselling on IFAS, knowledge of IFA supplementation, knowledge of anaemia, fourth visit for ANC and early registration for ANC in the section of methods. Different studies could have different definitions.

Thank you for the comment Reviewer #1. We gave reported issues you pointed out.

- **Knowledge on anemia** was defined as those who heard about anemia and know at least one of signs and symptoms of anemia,
- **Knowledge about IFAS** was measured by asking knowledge questions about IFAS (regarding knowledge of Iron/folate drug, health benefit of IFAS for the fetus and child, whether they believe taking IFAS has risks and whether they know for how long they should take the IFAS
- **Early registration to ANC** clinic was measured as those pregnant women who visit the ANC clinic before 16 weeks of gestation
- **Counseling on IFAS** was defined as Women who have received information on the IFA supplementation.
- **Fourth visit for ANC** was defined as pregnant women who received antenatal care (ANC) 4 or more times

5. Authors should present the results on characteristics and assessment of quality of study in main text not supplementary file because they are one of main results for meta-analysis.

Thank you for pointing out this useful concern. We agree that this statement. We have added characteristics of studies included as table 1 and Assessment of quality of studies as table 2 and was included in main test of the manuscript.

1. Due to too small sample size (study 39), maybe sensitive analysis is suggested when excluding this study.

Thank you for the comment Reviewer #1. As your suggestion Sensitivity analysis was performed during review and studies were removed from the review
7. Minor comments: for figure 2, the name of pooled rate should be added in the right place.

Thank you for the comment Reviewer #1. Considering your comment, Figure 2: was named “Forest plot displaying the pooled prevalence rate of compliance with IFAS among pregnant women in Sub-Saharan Africa”

Reviewer #2: 1. The background section of the abstract is a copy/paste of some introductory parts in the main manuscript. This is not a good practice. it is also very long and needs to shortened.

We appreciate your suggestion Reviewer #2. We revised background section of the abstract and shortened it.
2. Does anemia cause disability? This needs to be clarified and reference given.

We appreciate your suggestion Reviewer #2. As you raised issues of anemia cause disability; I have tried to review evidences and the following is some of it. Anemia affects different people in different ways. For those who have had a gradual reduction in red blood cells, their body may be able to handle the loss even if their red blood cell count is very low. Alternately, those who lose red blood cells quickly may show significant impairments. Those with illnesses that are already present, especially heart problems, may find that anemia makes their condition worse. And, of course, the severity of the anemia, and whether it responds to treatment, is key in determining whether anemia is disabling; for instance, severely low hemoglobin levels requiring frequent blood transfusions rises to the level of a disability

3. The definition of compliance is completely missing. This need to be included at appropriate section or subsection. Was the definition uniform across all the publication included for meta-analysis?

Thank you for the comment Reviewer #2. We have defined compliance in outcome interest section. Accordingly good compliance was considered as a pregnant woman who took ≥65% of the total prescribed IFA supplementation per month; whereas the opposite is true for non-compliance. Therefore, only articles defined in this way as included in meta-analysis.

4. Why was the DHS data not used? DHS data collection is uniform across countries and would have been the most suitable. The authors need to explain why DHS data was not used or was excluded from this analysis.

Thank you for the comment Reviewer #2. We removed DHS and Large survey data during sensitivity analysis because it over estimate the effect.

2. The conclusion both in the abstract and manuscript does not highlight on the future research opportunities and policy considerations. Only programmatic implications are highlighted.

Thank you for the comment Reviewer #2. Based on your point we have policy consideration and future research opportunities conclusion both in the abstract and manuscript.

.

6. The paper will benefit from review for grammar, typos and clarity.

Thank you for the comment Reviewer #2. We are also benefited from your review
