# Supplementary material for: Compliance with Iron and Folic Acid Supplementation (IFAS) and associated factors among pregnant women in Sub-Saharan Africa: A systematic review and meta-analysis
Source: PLoS One. 2021 Apr 14;16(4):e0249789. doi: 10.1371/journal.pone.0249789 (PMC8046188; doi:10.1371/journal.pone.0249789)
Supplement: Attachment — Submitted filename: A rebuttal letter second revision.docx [file pone.0249789.s006.docx]

Editors

PLOS ONE

December 4, 2021

Meseret Belete Fite

We thank you and the reviewers for a thorough reading and constructive criticism of our manuscript and for the opportunity to revise and resubmit. We are pleased to submit the improved research article “Compliance with Iron and Folic Acid Supplementation (IFAS) And Associated Factors Among Pregnant Women Sub-Saharan Africa: A systematic review and meta-analysis” for your consideration in PLOS ONE. On the following pages, you will find our response to the editor and reviewer comments. On behalf of my co-authors, I thank you for your consideration of this resubmission. We appreciate your time and look forward to your response.

Meseret Belete Fite

**Author’s Responses Reviewers' comments**

Reviewers' comments:

Reviewers' comments:

Reviewer's Responses to Questions

**Comments to the Author**

1. If the authors have adequately addressed your comments raised in a previous round of review and you feel that this manuscript is now acceptable for publication, you may indicate that here to bypass the “Comments to the Author” section, enter your conflict of interest statement in the “Confidential to Editor” section, and submit your "Accept" recommendation.

Reviewer #1: All comments have been addressed

Reviewer #2: (No Response)

Reviewer#2: We appreciate your suggestion. As your argument, we have again tried to revise the manuscript point to points. Thus, we are benefited from your suggestion

2. Is the manuscript technically sound, and do the data support the conclusions?

The manuscript must describe a technically sound piece of scientific research with data that supports the conclusions. Experiments must have been conducted rigorously, with appropriate controls, replication, and sample sizes. The conclusions must be drawn appropriately based on the data presented.

Reviewer #1: Yes

Reviewer #2: Yes

3. Has the statistical analysis been performed appropriately and rigorously?

Reviewer #1: Yes

Reviewer #2: Yes

4. Have the authors made all data underlying the findings in their manuscript fully available?

The [PLOS Data policy](http://www.plosone.org/static/policies.action#sharing) requires authors to make all data underlying the findings described in their manuscript fully available without restriction, with rare exception (please refer to the Data Availability Statement in the manuscript PDF file). The data should be provided as part of the manuscript or its supporting information, or deposited to a public repository. For example, in addition to summary statistics, the data points behind means, medians and variance measures should be available. If there are restrictions on publicly sharing data—e.g. participant privacy or use of data from a third party—those must be specified.

Reviewer #1: Yes

Reviewer #2: Yes

5. Is the manuscript presented in an intelligible fashion and written in standard English?

PLOS ONE does not copyedit accepted manuscripts, so the language in submitted articles must be clear, correct, and unambiguous. Any typographical or grammatical errors should be corrected at revision, so please note any specific errors here.

Reviewer #1: Yes

Reviewer #2: No

Thank you for the comment Reviewer #2. We appreciate the effort you made to enrich our manuscript more. Therefore, considering your comment, we used the skilled and an expert of English language to look at the language issues. Thus, we again revised by correcting any typographical or grammatical errors

6. Review Comments to the Author

Please use the space provided to explain your answers to the questions above. You may also include additional comments for the author, including concerns about dual publication, research ethics, or publication ethics. (Please upload your review as an attachment if it exceeds 20,000 characters)

Reviewer #1: Authors have addressed most of my concerns, and the manuscript has been improved. I have not addtional comments.

Reviewer #2: It would be good to explain in the resubmission how each comment has been addressed. For instance:
This comment has not been addressed: 'The background section of the abstract is a copy/paste of some introductory parts in the main manuscript. This is not a good practice. it is also very long and needs to shortened' the opening statement of the background still reads the same as the statement in the introduction

The definition of compliance is completely missing. This need to be included at appropriate section or subsection. Was the definition uniform across all the publication included for meta-analysis? WHERE IS THE OPERATIONAL DEFINITION OF COMPLIANCE?

The paper will STILL benefit from review for grammar, typos and clarity. THE TYPOS ARE STILL VISIBLE EVEN IN THE ABSTRACT

Thank you for the comment Reviewer #2. Hence, based on your suggestion, we revised the statements in the background section of the abstract; which is not directly taken from the main manuscript but similar concept. Also, we had shortened the background section of the abstract to only 47 words. However, regarding definition of compliance, we had included additional section which is “Operational definitions” and defined the concept of compliance here. Thus, all publication included in this meta-analysis were considered for this operational definition. Finally, as reviewer#2 suggestion, we were benefited from the comment in revision of the manuscript for grammar, typos and clarity. Thus, our deepest thanks go to Dr. Desalegn Luche a PHD Candidate and lecturer in English language at Haramaya University, Ethiopia; for his tirelessly supported and edited for the grammar, typos and clarity this manuscript.

7. PLOS authors have the option to publish the peer review history of their article ([what does this mean?](https://journals.plos.org/plosone/s/editorial-and-peer-review-process#loc-peer-review-history)). If published, this will include your full peer review and any attached files.


If you choose “no”, your identity will remain anonymous but your review may still be made public.


**Do you want your identity to be public for this peer review?** For information about this choice, including consent withdrawal, please see our [Privacy Policy](https://www.plos.org/privacy-policy).

Reviewer #1: No

Reviewer #2: No

[NOTE: If reviewer comments were submitted as an attachment file, they will be attached to this email and accessible via the submission site. Please log into your account, locate the manuscript record, and check for the action link "View Attachments". If this link does not appear, there are no attachment files.]

While revising your submission, please upload your figure files to the Preflight Analysis and Conversion Engine (PACE) digital diagnostic tool, <https://pacev2.apexcovantage.com/>. PACE helps ensure that figures meet PLOS requirements. To use PACE, you must first register as a user. Registration is free. Then, login and navigate to the UPLOAD tab, where you will find detailed instructions on how to use the tool. If you encounter any issues or have any questions when using PACE, please email PLOS at [figures@plos.org](mailto:figures@plos.org). Please note that Supporting Information files do not need this step.
